# Survival of Five Strains of Shiga Toxigenic* Escherichia coli* in a Sausage Fermentation Model and Subsequent Sensitivity to Stress from Gastric Acid and Intestinal Fluid

**DOI:** 10.1155/2017/5176384

**Published:** 2017-10-29

**Authors:** Tone Mari Rode, Anette McLeod, Ingrid Måge, Even Heir, Lars Axelsson, Askild L. Holck

**Affiliations:** ^1^Nofima AS-Norwegian Institute of Food, Fisheries and Aquaculture Research, P.O. Box 8034, 4068 Stavanger, Norway; ^2^Nofima AS-Norwegian Institute of Food, Fisheries and Aquaculture Research, P.O. Box 210, 1431 Ås, Norway

## Abstract

The ability of foodborne pathogens to exhibit adaptive responses to stressful conditions in foods may enhance their survival when passing through the gastrointestinal system. We aimed to determine whether* Escherichia coli* surviving stresses encountered during a model dry-fermented sausage (DFS) production process exhibit enhanced tolerance and survival in an* in vitro* gastrointestinal model. Salami sausage batters spiked with five* E. coli* isolates, including enterohaemorrhagic* E. coli* strains isolated from different DFS outbreaks, were fermented in a model DFS process (20°C, 21 days). Control batters spiked with the same strains were stored at 4°C for the same period. Samples from matured model sausages and controls were thereafter exposed to an* in vitro* digestion challenge. Gastric exposure (pH 3) resulted in considerably reduced survival of the* E. coli* strains that had undergone the model DFS process. This reduction continued after entering intestinal challenge (pH 8), but growth resumed after 120 min. When subjected to gastric challenge for 120 min,* E. coli* that had undergone the DFS process showed about 2.3 log_10_⁡ lower survival compared with those kept in sausage batter at 4°C. Our results indicated that* E. coli* strains surviving a model DFS process exhibited reduced tolerance to subsequent gastric challenge at low pH.

## 1. Introduction

In their natural habitats,* Enterobacteriaceae* are constantly under assault from different environmental stresses. One of the most frequently encountered hostile conditions is acid stress. While travelling through the gastrointestinal tract, bacteria must endure low pH conditions in the stomach, and the ability of foodborne pathogens to exhibit adaptive responses to stressful conditions in foods may enhance their survival.

Shiga toxigenic* Escherichia coli* (STEC) are potential foodborne pathogens. A STEC subgroup, enterohaemorrhagic* E. coli* (EHEC), is responsible for severe illness in humans and their infectious dose can be as few as 1–100 bacteria [1, 2]. EHEC may survive in a range of foods [3] and in the harsh environment of the gastrointestinal tract [4]. Currently, there is no specific treatment for EHEC infections, but supportive therapy is available. The use of conventional antibiotics may worsen Shiga toxin-mediated cytotoxicity [5]. Isolates belonging to the serotype O157:H7 were for many years the most commonly reported agents of EHEC infections, but non-O157:H7 STEC serotypes are increasingly being reported [6–8].

There have been several STEC outbreaks linked to dry-fermented sausages (DFS) in which different serotypes were reported as the infectious agent [9–12]. In DFS production, combinations of salt, nitrite, starter culture, lactic acid, low pH, and drying are used as hurdles to inhibit and reduce survival of pathogens [13]. However, studies have shown that in spite of exposure to unfavourable conditions like high NaCl concentrations and an acidic environment in DFS,* E. coli* O157:H7 can still survive [14–16]. Although there is variation between* E. coli* strains, certain EHEC strains within the serotypes O157:H7 and O104:H4 are more acid resistant than generic* E. coli* strains [17, 18].

We previously investigated strain dependent reductions of 11* E. coli* isolates in the DFS production process and during relevant postprocess treatments of DFS [19]. The results showed varying reductions between 1.3 and 2.4 log_10_⁡cfu g^−1^ for the* E. coli* strains during the sausage production process. Different postprocess treatments like storage, heating, and freezing gave additional reductions [19–21]. In the present work, we investigate whether* E. coli* surviving the stresses encountered during a model DFS production process, a tube fermented sausage (TFS) production, would exhibit enhanced tolerance in a gastrointestinal* in vitro* model. We added EHEC to a popular Norwegian DFS salami batter used in previous investigations [19–22] and, following TFS production, bacteria were exposed to digestion challenge.

## 2. Materials and Methods

### 2.1. Bacterial Isolates and Growth Conditions

Isolates of* E. coli* included five outbreak strains of different serotypes with varying* stx*-profiles, of which four strains were EHEC (Table 1), also used in a previous study by Rode et al. [19]. The strains were maintained at −80°C in tryptic soy broth (TSB; Oxoid, Thermo Fisher Scientific, Basingstoke, UK) supplemented with 20% glycerol (v/v). Prior to experiments, the* E. coli* strains were cultured separately in TSB for 16–18 h at 37°C, in a shaking incubator (200 rpm), and then stored at 4°C for 20 h. The strains used in TFS model experiments were added to sausage batter at 10^6^–10^7^ cfu g^−1^. Freeze-dried starter culture LS-25 (*Lactobacillus sakei* and* Staphylococcus carnosus* in a 1 : 1 mixture; Gewürzmüller, GmbH, Germany) was resuspended in 0.9% NaCl, at 4°C just prior to adding the starter culture mix to give a total level of 10^6^ cfu g^−1^ to the batters.

### 2.2. Tube Fermented Sausage Model

Sausage batter was prepared and fermented* in vitro* using sterile tubes mainly as described by Heir et al. [20]. In short, the batter contained meat from beef and pork (37.8% each) and lard from pork (20%). One bulk of sausage batter was made for the experiments, from which 2-kg packages were vacuum packed and stored at −20°C. On the day of sausage production, slightly thawed batter was supplemented with NaCl, NaNO_2_, and dextrose to give final concentrations of 3.8% NaCl, 100 ppm NaNO_2_, and 0.9% dextrose in the batter. Starter culture LS-25 was added to half of the batter. Each of the* E. coli* strains was individually added to aliquots of batters with and without starter culture. A rotating bowl kitchen machine was used for successively mixing ingredients and bacterial culture into the batter. Aliquots of 30 g of prepared sausage batter were transferred to 50-ml sterile centrifuge tubes (VWR, Radnor, PA, USA), thereby named “tube fermented sausages (TFS),” and centrifuged at 600*g* for 2 min to compress the batter and avoid air pockets. The sausage batters containing LS-25 were incubated at 20°C for 21 days (fermentation period), followed by storage at 4°C for 24 h, while control batters without LS-25 were incubated at 4°C for 22 days. The 24 h cooling period was included to avoid confounding effects caused by differences in temperature for the* E. coli* cells in the fermented batter compared with the control batter. Using this TFS model, the fermented sausage batters obtained an average water activity (*a*_w_) of approx. 0.95 [20]. Three productions were performed on different days, each including two parallel batter samples for each* E. coli* isolate. This resulted in three sets of 20 samples (2 sample types (fermented and controls), 2 parallels, and 5 strains).

### 2.3. Microbial and Physiochemical Analyses

At days 0 and 22, samples (15 g) from matured TFSs and from controls were diluted 1 : 10 (w/v) in peptone water and homogenized for 1 min in a stomacher (AES Smasher, AES Chemunex, Bruz, France). Quantification of* E. coli* was performed using a mechanical spiral plater (Whitley Automatic Spiral Plater, Don Whitley Scientific Ltd., West Yorkshire, UK) on tryptic soy agar (TSA, Oxoid) for 16 h. The TSA plates were incubated at 42.5°C to prevent growth of the starter culture and the indigenous flora of the meat batter. Lack of growth of the starter culture and the indigenous flora at this temperature was confirmed in previous studies [19]. Lactic acid bacteria were plated on MRS agar (Oxoid) for 48 h at 30°C to verify the activity of the starter culture. Manual plating was used for samples with low concentrations of bacteria. The detection limit was 20 cfu g^−1^ batter. Counts of* E. coli* and starter culture were determined individually from each sample. The probability of isolating confounding indigenous subpopulations of* E. coli* and other* Enterobacteriaceae* during the experiment was assumed low because prior studies showed these organisms were present at levels of several log_10_⁡ values below those of the inoculated STEC strains [19]. Furthermore, the indigenous flora failed to grow under the experimental conditions (42.5°C) used to cultivate the STEC strains (data not shown). Subtyping (serotype) the* E. coli* isolates recovered from the meat batters was therefore not performed. pH was measured in duplicate in stomacher-homogenized solutions used for microbiological analysis during fermentation at days 0, 1, 2, 3, 5, 7, 8, 10, 11, 12, 14, 15, 18, 20, and 22. The pH was also measured at selected time points during the digestion challenge.

### 2.4. Digestion Challenge Model

**Figure 1:**
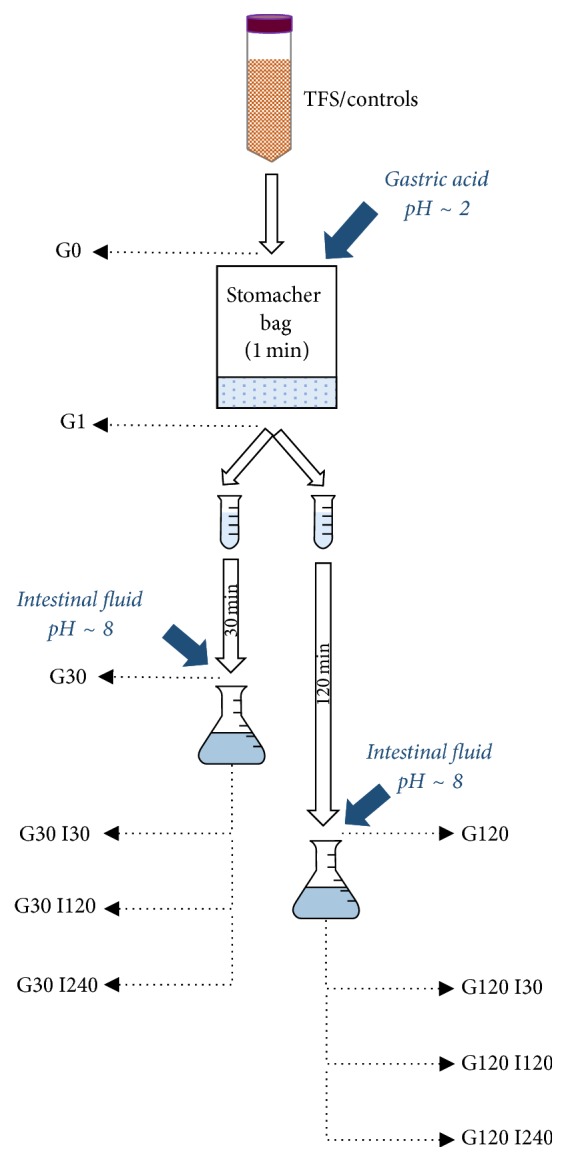
Flow chart illustrating the experimental setup. TFS (tube fermented sausage) and control batter (15 g) were transferred to separate stomacher bags, diluted 1 : 10 in gastric acid solution, and stomached for 1 min. Samples were transferred to tubes and incubated for 1, 30, and 120 min (samples G1, G30, and G120, resp.). Furthermore, intestinal fluid solution was added to samples after 30 and 120 min (1 : 1). Sampling from G30 and G120 tubes was performed after 30, 120, and 240 min. Each experiment was repeated three times and included 2 sample types (fermented and controls) × 2 parallels × 5* E. coli* strains. A total of 60 (3 × 20) samples were included for the digestion challenge study.

The matured TFSs and controls were exposed to gastric acid (G) and intestinal fluid (I) in an experimental design as listed in Table 2 and illustrated in Figure 1. The gastric acid solution was prepared as described by Molly et al. [23] by mixing the following ingredients: 3.0 g l^−1^ yeast extract; 1.0 g l^−1^ Bacto peptone (Difco, Detroit, USA); 0.5 g l^−1^ cysteine; 0.4 g l^−1^ glucose; 4.0 g l^−1^ porcine mucin; 0.08 g l^−1^ NaCl; 0.4 g l^−1^ NaHCO_3_; 0.04 g l^−1^ K_2_HPO_4_; 0.04 g l^−1^ KH_2_PO_4_; 0.008 g l^−1^ CaCl_2_·2H_2_O; 0.008 g l^−1^ MgSO_4_·7H_2_O; 1.0 g l^−1^ xylan; 3.0 g l^−1^ soluble starch; 2.0 g l^−1^ pectin; and 1 ml l^−1^ Tween 80. The solution was autoclaved and cooled, and then 3 g l^−1^ pepsin from porcine stomach mucosa (Sigma-Aldrich, Steinheim, Germany) was added. By using 10 mol l^−1^ HCl, the pH was adjusted to 2.0. The intestinal fluid solution was prepared fresh by mixing 0.25 g l^−1^ porcine pancreatin (Sigma-Aldrich) and 3 g l^−1^ porcine bile and was filtrated (0.45 *μ*m, Nalgene, Rochester, USA) before use [24]. Samples were kept at 37°C during the digestion challenge experiments. Tube fermented sausage batters (15 g) were transferred to separate stomacher bags, diluted 1 : 10 by addition of 135 ml gastric acid solution, and stomached. Samples were incubated for 1, 30, and 120 min simulating different duration of exposure to gastric acid (samples G1, G30, and G120, resp.; Figure 1 and Table 2). Furthermore, 20 ml intestinal fluid solution was added to 20 ml samples of G30 and G120 (1 : 1), and pH was adjusted to 8 using 5 mol l^−1^ NaOH. Sampling from G30 and G120 tubes to which intestinal fluid was added was then performed after 30, 120, and 240 min (I30, I120, and I240, resp.; Table 2). The G1 samples were used to measure the immediate response to gastric acid exposure. After the digestion challenge experiments, samples were immediately subjected to microbial analysis (described above). Control batters were treated in a similar matter as the TFS.

### 2.5. Statistical Analysis

*E. coli* reductions between time points *t*_0_ and *t*_1_ were calculated as log_10_⁡(*C*_*t*0_/*C*_*t*1_), where *C* is the counts of* E. coli* (cfu g^−1^). Analysis of variance (ANOVA) was used to determine statistically significant differences in* E. coli* reductions in various stages of the digestion challenge.

*(1) Gastric Treatments*.* E. coli* reductions between matured TFSs or controls (*t*_0_ = G0/day 22) and gastric acid incubation time (*t*_1_ = G1, G30, or G120 min) were analyzed with respect to the experimental factors “strain,” “fermentation,” and “gastric acid incubation time.”

*(2) Intestinal Treatments*.* E. coli* reductions between end of gastric treatments (*t*_0_ = G30 or G120) and intestinal fluid incubation time (*t*_1_ = I30, I120, or I240 min) were analyzed with respect to the experimental factors “strain,” “fermentation,” “gastric acid incubation time,” and “intestinal fluid incubation time.”

*(3) Digestion Time Lapse*. For each of the four groups “fermented-G30,” “fermented-G120,” “control-G30,” and “control-G120,” the differences between subsequent time points in the digestion process were analyzed. 

In all cases, a nested mixed model was used to calculate the ANOVA. Tubes (modelled as a random factor) are nested within fixed factors “strain” and “fermentation.” The factors “gastric acid incubation time” and “intestinal fluid incubation time” are within-tube fixed factors. Models included main effects and two-level interaction effects. The analyses were performed using MATLAB (R2014b, The Mathworks, Inc., Natick, USA, https://www.mathworks.com) and Minitab® Statistical Software (version 17.2.1, http://www.minitab.com).

## 3. Results

### 3.1. Reduction of* E. coli* in the TFS Model

**Figure 2:**
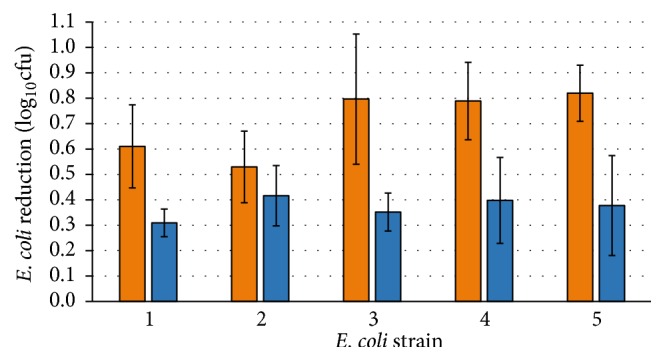
Reduction of* E. coli* in a TFS model. Salami batter with starter culture fermented at 20°C for 21 days giving matured sausages (orange bars) and meat batter controls without starter culture held at 4°C (blue bars) are shown. Isolates are numbered according to Table 1.

Results from matured TFS, batter with starter culture fermented at 20°C for 21 days, and 4°C controls are presented in Figure 2. The TFS production process resulted in a 0.7 log_10_⁡cfu g^−1^ average reduction of* E. coli*, ranging from 0.5 to 0.8 log_10_⁡cfu g^−1^, a small difference of only 0.3 log_10_⁡ between the most and least resistant isolates, 2 and 5, respectively. During the 21-day sausage production period, the pH rapidly dropped from 5.7 to 4.6 within two days and then remained stable. At the end of the period, the average pH was 4.63 ± 0.05 (range 4.57–4.71). For the corresponding 4°C controls, lower* E. coli* reductions were observed, ranging from 0.3 to 0.4 log_10_⁡, and the pH remained at 5.7 for 14 days before slowly declining to an average pH of 4.97 ± 0.17 at the end of the period.

### 3.2. Reduction of* E. coli* during Digestion Challenge

**Figure 3:**
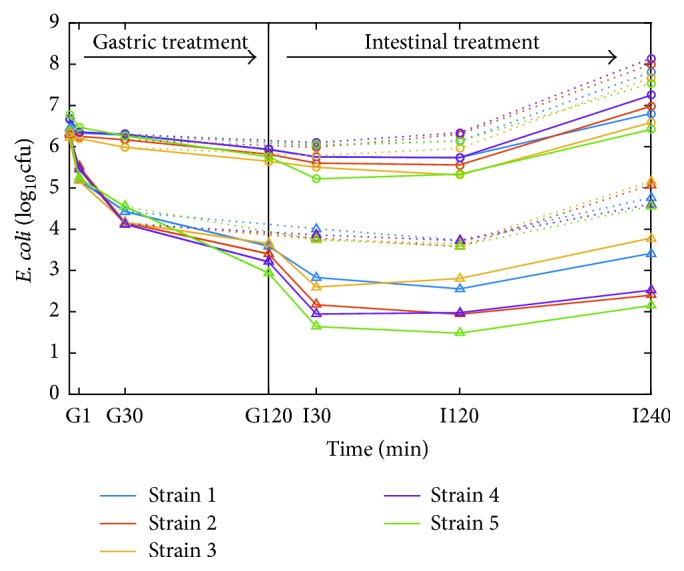
Counts of* E. coli* during digestion challenge. Salami batter with starter culture fermented at 20°C for 21 days and thereafter stored for 24 h at 4°C (∆) and control batters without starter culture which were held at 4°C for 22 days (○) are shown. Exposure of samples to gastric acid (gastric treatment; G) for 30 or 120 min and subsequently to intestinal fluid (intestinal treatment; I) for 240 min is according to Table 2. Dotted and continuous lines represent samples exposed to 30 and 120 min of gastric treatments, respectively, before intestinal treatment. Average values from three independent experiments with two parallels each are given, and strains are numbered according to Table 1.

Reductions of* E. coli* in the TFS samples were significantly larger (*p* < 0.001) during gastric acid treatments compared with controls (Figure 3, Tables 2 and 3). Already after 1 min (G1), the five* E. coli* strains showed an average reduction of 1.0 log_10_⁡ (range 0.8–1.3) in the TFS samples. Continued reduction was seen after 30 min, with an average reduction of 2.1 log_10_⁡ (range 1.8–2.2), which after 120 min averaged 3.0 log_10_⁡. For the 4°C controls, the average reduction was only 0.2 log_10_⁡ after 1 min of gastric acid treatment. Although at a low level, continued reductions were thereafter seen both from 1 to 30 min and from 30 to 120 min of gastric acid treatment, with log_10_⁡ values of 0.4 and 0.7 log_10_⁡, respectively. The pH during gastric challenge ranged from 2.88 to 3.21 for all TFS and controls, where the TFS samples had an average pH of 3.10 ± 0.12, and the control samples had marginally lower value of 3.01 ± 0.11 (*p* < 0.05).

For the TFS samples exposed to the longest acid stress treatment lasting for 120 min (G120), continued reduction was seen until 30 min in intestinal fluid (*p* < 0.001) (Table 4), reaching an average of 4.1 log_10_⁡ (range 3.6–4.7). After 30 and 120 min in intestinal fluid, cell counts remained unchanged (*p* > 0.05). Furthermore, the bacterial cells seemed to recover, as growth was observed from 120 to 240 min in intestinal fluid, and the average reduction was 3.5 log_10_⁡ (range 2.4–4.2) at the end of the experiment.* E. coli* in the 4°C controls exposed to gastric acid for 120 min showed an average reduction of 1.0 log_10_⁡ (range 0.7–1.6) after 30 min in intestinal fluid (*p* < 0.001). No further reduction was seen between 30 and 120 min in intestinal fluid (*p* > 0.05), and the average reduction remained 1.0 log_10_⁡ (range 0.8–1.4) after 120 min. From 30 to 240 min in intestinal fluid, the bacterial cells in the controls seemed to recover and started growing. Specifically, from 120 to 240 min in intestinal fluid, the cells multiplied and reached higher numbers than before digestion challenge (*p* < 0.001).

*E. coli* in the TFS samples exposed to the shorter gastric acid treatment lasting for 30 min (G30) showed only slight additional reduction after subsequent 30 min in intestinal fluid (*p* < 0.001), with an average reduction of 2.5 log_10_⁡ (range 2.4–2.6). Between 30 and 120 min in intestinal fluid, no further reduction occurred (*p* > 0.05), and the bacterial cells seemed to recover. From 120 to 240 min in intestinal fluid, there was an increase in bacterial numbers and the average reduction was only 1.5 log_10_⁡ (range 1.1–1.8) at the end of the experiment. For* E. coli* in the 4°C controls exposed to acid stress for 30 min, a small reduction was seen after subsequent 30 min in intestinal fluid (*p* < 0.01), with an average of 0.6 log_10_⁡ (range 0.3–0.7). From 30 and 120 min in intestinal fluid, the cells recovered and started to grow, and from 120 to 240 min, cell counts were higher than before digestion challenge.

The fermentation process was found to have the largest impact on reductions of* E. coli* in the gastric acid treatment (Table 5). In other words, bacterial reduction differed the most between matured TFSs and corresponding controls. Changing the duration of gastric acid treatment also had a large effect, and there was an interaction effect between fermentation and gastric acid treatment duration. The duration of intestinal fluid treatment had largest effect on bacterial reduction in the intestine (Table 6). There were also individual effects of fermentation and of gastric incubation time and an interaction effect between treatment duration with intestinal fluid and fermentation.

ANOVA on the results from matured TFSs and batter controls separately demonstrated statistically significant variations in bacterial reductions between the different* E. coli* strains, though the variations were small (results not shown). Considering gastric acid treatments,* E. coli* reductions in controls treated for 120 min showed a 0.5 log_10_⁡ difference between strains 2 and 5. The largest strain variation was observed for the TFS samples exposed to gastric acid for 120 min followed by 240 min in intestinal fluid (G120I240), where a 1.8 log_10_⁡ difference was seen between strains 3 and 5 (reductions of 2.4 log_10_⁡ and 4.2 log_10_⁡, resp.). Furthermore, there were no strain differences for the TFSs exposed to gastric acid for 30 min and subsequently intestinal fluid for 240 min (G30I240). For the corresponding controls exposed to gastric acid for 30 min followed by 240 min in intestinal fluid (G30I240), the strains grew well and average counts ended with 1.7 log_10_⁡ higher than before the intestinal challenge, where a statistically significant difference was seen in strains 2, 3, and 4 recovering better than strain 5.

## 4. Discussion

We aimed to examine how* E. coli* outbreak strains of different serotypes subjected to a fermented sausage production process survive a subsequent gastric and intestinal challenge. Our hypothesis was that strains adapted to acid during the production process might show enhanced survival in digestion challenge. The effect of fermentation (at 20°C) and low pH (4.6) in a fermented sausage model (tube fermented sausages, TFS) on the survival of* E. coli* was compared with bacterial survival in sausage batter stored at 4°C (control). In previous studies, parameters of tube fermented sausages were similar to those of conventional fermented sausages containing the same meat matrix with regard to NaCl concentration, pH development, and lactic acid production [20, 25]. Thus we consider the TFS model useful for the gastrointestinal challenge experiments even though very limited drying occurs during the tube fermentation process.

The resulting data from TFSs and control batters exposed to the* in vitro* digestion challenge model showed a marked difference in* E. coli* survival between the two. ANOVA models were useful for determining the statistically significant effects on* E. coli* reduction. Contrary to what we initially expected,* E. coli* undergoing TFS production at 20°C and pH 4.6 showed higher reduction when subjected to gastric challenge (2.1 and 3.0 log_10_⁡ after 30 and 120 min, resp.), compared with* E. coli* in control sausage batter at 4°C and pH 5.0 (Figure 2). The fermented meat samples were diluted tenfold with simulated gastric juice. Although diluted, the samples still contained a low amount of lactic acid. Since the pH was low, the majority of this lactic acid would be in undissociated form able to penetrate the cell membrane and contribute to acid stress. Control samples stored at 4°C also underwent a slow spontaneous fermentation process from day 14 and reached a pH of 5.0 by day 22; thus undissociated lactic acid would also present in these samples during the gastric challenge. Since the fermented samples and the controls had similar pH during gastric challenge and both contained undissociated lactic acid, the enhanced reduction in survival is likely caused by the influence of the overall fermentation process for the 20°C matured TFS. After incubation in intestinal fluid, reduction of bacterial cells continued up to 30 min, with a more pronounced reduction for the cells that had undergone the TFS process. Likely, this reflects that increasing cellular damage was inflicted with increasing duration of the gastric acid exposure. However, the lag time before growth commenced appeared to be fairly similar for cells surviving for 30 and 120 min in the acidic environment, and cells grew well in all samples after recovery, regardless of previous treatment.

In contrast to our findings, Naim et al. [24] previously demonstrated that* E. coli* O157:H7 isolates surviving a dry-fermented sausage process acquired a strong protective effect and survived in the digestive fluids. The average pH differed between their findings and ours. During gastric acid treatment, the pH in our study was 3.05, whereas Naim et al. [24] demonstrated a pH of 3.20. Moreover, their target pH after fermentation was 4.9, compared with 4.6 in our study. This pH difference likely account for some of the differences seen in* E. coli* survival between the two studies. A fermentation of summer sausages to pH 4.6 and pH 5.0, followed by mild heat treatment, was previously shown by Calicioglu et al. [26] to give a reduction of* E. coli* O157:H7 of ≥7.0 and 3.2 log_10_⁡, respectively. This could indicate that even small changes in the final pH in a fermented product have a large impact on bacterial survival when exposed to further stress. When pH was increased to 8 (intestinal challenge), there was an additional reduction before a recovery and growth initiation was observed for the strains in our study. This recovery pattern was partly different from findings by Naim et al. [24] where* E. coli* remained stable after the passage to the intestinal challenge. However, in both studies, growth was observed after 120 min.

Several reports have stated that different* E. coli* isolates vary widely in their ability to survive low pH conditions [15, 27, 28], while others have claimed that O157 strains have higher acid tolerance compared with strains of other* E. coli* serogroups [17, 27, 29, 30]. In our present study, which included both O157:H7 and O157:H- and outbreak isolates from serogroups O103 and O111, the non-O157 isolates had the same reduction profile as the O157 isolates. Our former investigation also demonstrated similar survival of the O157 and non-O157 isolates after storage in DFS at 4, 16, and 20°C for 1, 2, and 3 months [19]. Bergholz and Whittam [29] studied the impact of acidity using STEC strains including O157:H7, O26:H11, and O111:H8 inoculated in apple juice stored at 4 and 22°C for 24 h prior to gastric challenge. The prestorage at 4°C resulted in higher bacterial survival than prestorage at 22°C, and the mean survival rate of the O157:H7 strains was more than three times higher compared with O26 and O111 isolates. Storage at low temperature in our present study also gave higher survival of* E. coli* at low pH, although there was no higher tolerance of the tested* E. coli* serogroup O157 strains. In a large meta study by McQuestin et al. [31], temperature was stated to have the largest impact on inactivation of* E. coli* during fermentation in meat.

When bacteria are exposed to stress, they can enter a viable, nonculturable condition. Injured cells can enter this state. Severe stress as a consequence of exposure to food matrices and high or low temperature can lead to increased cell injury and decreased bacterial survival. The reduction numbers from the TFSs are based on growth on agar plates at 42.5°C; thus it cannot be ruled out that some injured cells might have had difficulties in growing at this temperature. However, in our previous investigations, some of the strains were plated under various conditions for recovering injured cells, but we did not discover any viable, nonculturable cells [19].

## 5. Conclusions

We have shown that* E. coli* surviving a model tube fermented sausage (TFS) process exhibit reduced tolerance to low pH in a subsequent digestion challenge model due to the extended exposure to acidic conditions and storage at ambient temperature during sausage fermentation. The* E. coli* O157 isolates tested had a survival pattern similar to the non-O157 isolates when exposed to the environment in the digestive system, but the limited number of strains and their origins being connected to DFS restrict us from concluding whether they have similar abilities to endure acid stress. Investigating a larger selection of strains of various origins and serotypes could aid in determining this. Further studies should also include various sausage fermentation and digestion challenge conditions to widen the knowledge of the role of DFS process parameters in reducing microbial food safety risks of this type of products.

## Figures and Tables

**Table 1:** *E. coli* isolates used in this study.

Number	Strain	Serotype	*stx1*	*stx2*	Source	Comments/reference
(1)	E218/02	O157:H7	−	+	Dry-fermented sausage	Outbreak Sweden, 2002^*∗*^ [11]
(2)	MF3582	O157:H-	−	+	Human, clinical	Outbreak Norway 2009^†^, sorbitol positive [19]
(3)	MF2411	O111:H-	+	+	Semidry-fermented sausage (mettwurst)	Outbreak Australia, 1995^‡^ [10]
(4)	MF2494	O103:H25	−	+	Human, clinical	Outbreak Norway 2006^§^ [12]
(5)	MF2522	O103:H25	−	−	Dry-fermented sausage (morr)	Linked to outbreak in Norway, 2006^§^ [12]

^*∗*^Kindly received from Dr. S. Löfdahl, Swedish Institute for Infectious Disease Control, Solna, Sweden. ^†^Kindly received from Professor G. Kapperud, Norwegian Institute of Public Health, Oslo, Norway. ^‡^Kindly received from Dr. F. Scheutz, Statens Serum Institut, Copenhagen, Denmark. ^§^Kindly received from Dr. C. Sekse, Norwegian School of Veterinary Science, Oslo, Norway.

**Table 2:** Digestion challenge model treatments^*∗*^.

Sample	Treatment time (min)	
	Gastric acid	Intestinal fluid
G1	1	0
G30	30	0
G30I30	30	30
G30I120	30	120
G30I240	30	240
G120	120	0
G120I30	120	30
G120I120	120	120
G120I240	120	240

^*∗*^Details are described in Materials and Methods. *Digestion challenge model*. *E. coli* isolates surviving a TFS production process were exposed to a model mimicking part of the gastrointestinal tract. G: gastric acid treatment and I: intestinal fluid treatment.

**Table 3:** Reduction of *E. coli* during gastric treatment^*∗*^.

Fermentation status	Strain	Gastric treatment time (min)		
		1	30	120
TFS	(1)	1.26 (0.20)	2.03 (0.26)	2.87 (0.63)
	(2)	0.76 (0.30)	2.12 (0.27)	2.88 (0.32)
	(3)	1.04 (0.50)	2.05 (0.60)	2.56 (0.65)
	(4)	0.88 (0.48)	2.22 (0.24)	3.14 (0.14)
	(5)	1.14 (0.35)	1.84 (0.64)	3.45 (0.46)
Control	(1)	0.32 (0.17)	0.37 (0.16)	0.71 (0.13)
	(2)	0.06 (0.02)	0.14 (0.09)	0.49 (0.05)
	(3)	0.13 (0.13)	0.35 (0.12)	0.69 (0.24)
	(4)	0.32 (0.08)	0.38 (0.18)	0.75 (0.15)
	(5)	0.30 (0.18)	0.51 (0.31)	1.02 (0.20)

^*∗*^The numbers are average reductions of log_10_⁡cfu values compared with before gastric treatment. Standard deviation values are shown in brackets.

**Table 4:** Reductions of *E. coli* during intestinal treatment^*∗*^.

Fermentation status	Strain	Intestinal treatment time after 30 min gastric treatment (min)			Intestinal treatment time after 120 min gastric treatment (min)		
		30	120	240	30	120	240
TFS	(1)	0.42 (0.38)	0.70 (0.26)	−0.33 (0.36)	0.76 (0.16)	1.03 (0.79)	0.18 (0.64)
	(2)	0.37 (0.34)	0.58 (0.22)	−0.91 (0.51)	1.23 (0.14)	1.46 (0.16)	1.00 (0.86)
	(3)	0.41 (0.22)	0.50 (0.22)	−1.00 (0.18)	1.05 (0.16)	0.85 (0.46)	−0.13 (0.35)
	(4)	0.26 (0.38)	0.40 (0.39)	−0.49 (0.43)	1.27 (0.53)	1.23 (0.49)	0.69 (0.90)
	(5)	0.80 (0.76)	0.95 (1.35)	−0.01 (1.28)	1.30 (0.20)	1.46 (0.30)	0.79 (0.43)
Control	(1)	0.25 (0.19)	0.14 (0.14)	−1.52 (0.28)	0.18 (0.22)	0.20 (0.14)	−0.86 (0.34)
	(2)	0.18 (0.24)	−0.13 (0.14)	−1.83 (0.09)	0.22 (0.28)	0.26 (0.12)	−1.16 (0.55)
	(3)	0.20 (0.20)	0.03 (0.18)	−1.69 (0.30)	0.15 (0.42)	0.33 (0.36)	−0.94 (0.46)
	(4)	0.20 (0.08)	−0.04 (0.09)	−1.84 (0.13)	0.18 (0.16)	0.20 (0.10)	−1.32 (0.16)
	(5)	0.21 (0.18)	0.13 (0.07)	−1.27 (0.44)	0.54 (0.41)	0.42 (0.12)	−0.66 (0.49)

^*∗*^The numbers are average reductions of log_10_⁡cfu values compared with after gastric treatment. Standard deviation values are shown in brackets.

**Table 5:** ANOVA of *E. coli *reductions during gastric acid treatment in a TFS model^†^.

Source	Degrees of freedom	Explained variance
Strain (*S*)	4	1.0
Fermentation (*F*)	1	56.3^*∗*^
Gastric acid incubation time (*G*)	2	22.9^*∗*^
*S* × *F*	4	0.1
*S* × *G*	8	1.0
*F* × *G*	2	8.1^*∗*^
Tube (within *F* and *S*)	50	6.0^*∗*^
Tube × *G* (within *F* and *S*)	100	3.8
Error	8	0.7
*R* ^2^_adjusted_		0.83

^†^Main effects and two-factor interactions are included. The factor tube is modelled as random, while all other factors are considered fixed. Numbers in the table correspond to explained variances (sum-of-squares as % of total sum-of-squares), and significant effects on 1% level are marked by *∗*. The model is based on gastric acid treatments for 1, 30, and 120 min (G1, G30, and G120, resp.; Table 2). Other factors are fermentation (4 or 20°C) and strain (*E. coli *isolates, Table 1).

**Table 6:** ANOVA of *E. coli* reductions during intestinal fluid treatments in a TFS model^†^.

Source	Degrees of freedom	Explained variance
Strain (*S*)	4	2.2
Fermentation (*F*)	1	21.6^*∗*^
Gastric incubation time (*G*)	1	8.3^*∗*^
Intestine incubation time (*I*)	2	35.8^*∗*^
*S* × *F*	4	0.8
*S* × *G*	4	0.7
*S* × *I*	8	0.5
*F* × *G*	1	1.5
*F* × *I*	2	2.7^*∗*^
*G* × *I*	2	1.4^*∗*^
Tube (within *F* and *S*)	50	8.2
Tube × *G* (within *F* and *S*)	50	7.5
Tube × *I* (within *F* and *S*)	100	4.7^*∗*^
Error	130	4.1
*R* ^2^_adjusted_		0.89

^†^Main effects and two-factor interactions are included. The factor tube is modelled as random, while all other factors are considered fixed. Numbers in the table correspond to explained variances (sum-of-squares as % of total sum-of-squares), and significant effects on 1% level are marked by *∗*. The model is based on intestinal treatments for 30, 120, and 240 min (I30, I120, and I240, resp.; Table 2) after 30 or 120 min of gastric acid exposure (G30 and G120, resp.; Table 2). Other factors are fermentation (4 or 20°C) and strain (*E. coli* isolates, Table 1).
